# From biophysical interaction to structural modeling: bi-terminal G and TGS domains drive rice OsYchF1-OsGAP1 complex formation

**DOI:** 10.1186/s40529-025-00480-0

**Published:** 2025-09-29

**Authors:** Suchang Huang, Ziting Wu, Kuang Wang, Yunchuan Liao, Jiaqiang Zeng, Rui Miao

**Affiliations:** Fujian Provincial Key Laboratory of Plant Functional Biology, College of Life Sciences, Fujian Agriculture and Forestry University, Fuzhou, 350002 P. R. China

**Keywords:** Unconventional g protein, OsYchF1, G domain, Helical domain, TGS domain, C2-domain OsGAP1, Yeast two-hybrid, Isothermal titration calorimetry, AlphaFold2, ClusPro 2.0

## Abstract

OsYchF1 belongs to the YchF subfamily in the Obg family of G proteins, and comprises a core G domain, an α-helical domain, and a TGS domain from the N-terminus to the C-terminus. The C2-domain *Oryza sativa* GTPase-activating protein (OsGAP1) binds to and accelerates the enzyme activity of OsYchF1. The OsYchF1–OsGAP1 module plays a pivotal role in the response of rice to environmental stress. Nonetheless, how OsYchF1 anchors OsGAP1 has remained elusive. Our results demonstrate that OsYchF1 interacts with OsGAP1 through its N-terminal G domain and C-terminal TGS domain, while the inserted helical domain imposes a steric hindrance, as shown by yeast two-hybrid and isothermal titration calorimetry assays. Also, we implemented the AlphaFold2 artificial intelligence system to predict the structure of the OsYchF1–OsGAP1 complex. However, AlphaFold2 could not capture these dynamic conformational changes, likely due to the weak (*K*_D_=102.6 ± 11.7 µM) and transient nature of the OsYchF1-OsGAP1 interaction, which is primarily driven by hydrophobic interactions. Unexpectedly, the protein-protein docking by a fully automated algorithm ClusPro accurately recapitulated both the experimental evidence for G/TGS domain-mediated binding and the known cluster regions on OsGAP1, whereas AlphaFold2 failed to align with these findings.

**Supplementary Information:**

The online version contains supplementary material available at 10.1186/s40529-025-00480-0.

## Introduction

Plants are sessile organisms, and must evolve sophisticated approaches to cope with environmental challenges (Miao et al. 2018, 2022). Phosphate-binding loop guanosine triphosphatases (P-loop GTPases), also known as guanine nucleotide-binding (G) proteins, are a class of critical signaling proteins found in most prokaryotes and eukaryotes (Verstraeten et al. 2011; Mohanasundaram and Pandey 2023). G proteins bind to guanosine triphosphate (GTP) and release guanosine diphosphate (GDP), thereby acting as binary molecular switches to regulate diverse life processes (Dohlman and Campbell 2019; Lin et al. 2023). Then, G proteins sense external stimuli and transduce them into an internal signaling network for responses (Wu and Lew, 2013 ).

On the one hand, G proteins can be classified into the Translation Association Factors (TRAFAC) superfamily and Signal Recognition Particle and the MinD and BioD (SIMIBI) superfamily according to amino acid sequence and structural characteristics (Koller-Eichhorn et al. 2007; Leipe et al. 2002). The TRAFAC superfamily has been associated with signal transduction, intracellular trafficking and translation regulation. By contrast, the SIMIBI superfamily is mainly involved in chromosome segregation, protein localization, membrane transport and regulation of metabolism (Mittenhuber et al., 2001; Cheung et al. 2010; Cheung et al. 2022). The universally conserved YchF subfamily is in the Obg family under the TRAFAC superfamily (Koller-Eichhorn et al. 2007; Leipe et al. 2002). On the other hand, G proteins are routinely composed of the small G proteins, the heterotrimeric G proteins and a group of unconventional G proteins (Assmann 2002; Luo et al. 2022; Lin et al. 2023). YchF proteins belong to an extremely conserved unconventional G protein existing in all kingdoms of life except algae (Gradia et al., 2009; Becker et al. 2012).

YchF orthologues are highly conserved across species, and are composed of a N-terminal G domain for nucleotide binding, an inserted large α-helical domain and a C-terminal TGS (Threonyl-tRNA synthetase (ThrRS), GTPase, and SpoT proteins) domain potentially for nucleic acid anchoring (Luo et al. 2022; Lin et al. 2023). Unlike other G proteins, YchF is flexible to both GTP and adenosine triphosphate (ATP), due to the unusual (N/T)(M/L/V)xE amino acid sequence of the G4 motif in the core G domain (Lin et al. 2023; Koller-Eichhorn et al. 2007; Cheung et al. 2016). Thus, YchF can bind and hydrolyze both GTP and ATP. *Oryza sativa* YchF (OsYchF1) (accession number NP_001390453.1) is a well-characterized rice homologue of the YchF subfamily. OsYchF1 is a negative regulator in the response of rice plants to biotic and abiotic stresses (Cheung et al. 2010, 2013, 2016). Currently, the only known interacting partner of OsYchF1 is the C2 domain-containing protein OsGAP1 (accession number NP_001403513.1) (Yung et al. 2015). OsGAP1 promotes OsYchF1 GTPase and ATPase activity, and acts as a positive regulator against environmental stress (Cheung et al. 2008, 2013; Cheng et al., 2022). Taken together, the OsYchF1-OsGAP1 module works as a molecular switch to mediate rice response to external stimuli (Cheung et al. 2008, 2013).

The binding of OsYchF1 to OsGAP1 represents a typical weak protein-protein interaction with a low binding affinity, and never enables co-crystallization and structure determination. So far, the Artificial Intelligence (AI) system AlphaFold2 developed by Google DeepMind and EMBL-EBI for protein structure prediction seems very efficient for a mechanistic and functional understanding of protein complexes. In addition, a few evolved programs (e.g., ClusPro 2.0, HADDOCK 3, et al.) help to derive the predicted model of protein complexes. In the current work, we first studied the interaction of OsYchF1 and its truncated versions with OsGAP1 by yeast two-hybrid assays and isothermal titration calorimetry measurements. Then, we utilized the ubiquitous unconventional G-protein rice YchF and its activator C2-domain OsGAP1 as a model of low-affinity binding complex to validate the predicted structures generated by AlphaFold2 and ClusPro. In summary, OsYchF1 interacts with OsGAP1 through the bi-terminal G and TGS domains. ClusPro provides accurate docking models for weak protein complexes but not AlphaFold2.

## Results

### OsYchF1 G and TGS domains are crucial for its interaction with OsGAP1

**Fig. 1:**
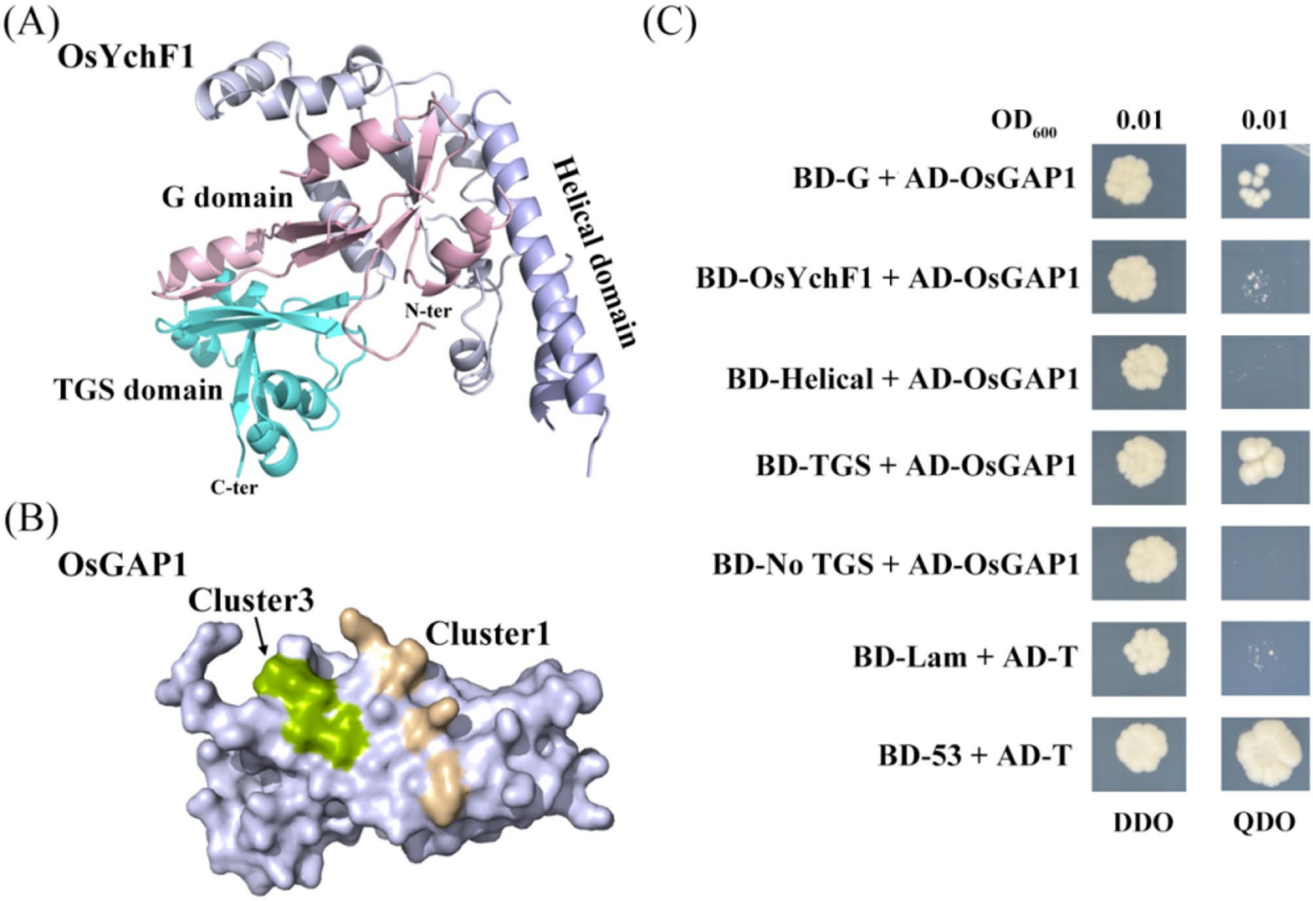
Yeast two-hybrid assay to monitor the interaction of full-length OsYchF1 and its truncated derivatives with OsGAP1. (**A**) Cartoon representation of the crystal structure of OsYchF1 (PDB code: 5EE0). The conserved N-terminal G domain (aa 1-128), the inserted helical domain (aa 141–199) and the C-terminal TGS domain (aa 303–394) of OsYchF1 are color-coded in pink, purple and cyan, respectively. (**B**) Cartoon representation of the structure of OsGAP1 (PDB code: 4RJ9). The indicated clusters 1 and 3 abolished the binding of OsGAP1 to OsYchF1. (**C**) Yeast two-hybrid assay to monitor the interaction of OsGAP1 with full-length OsYchF1, OsYchF1 G domain, helical domain, TGS domain and No TGS domain. The combination of BD-35 and AD-T was used as the positive control. The combination of Lam and AD-T was used as the negative control. OD_600_, optical density at 600 nm. DDO, double dropouts (SD/-Trp-Leu); QDO, quadruple dropouts (SD/-Trp-Leu-His-Ade); AD, the activation domain (prey); Lam, lamin; T, T-antigen; BD, the DNA binding domain (bait)

To investigate the OsYchF1–OsGAP1 complex, a yeast two-hybrid (Y2H) assay was initially employed to monitor the interaction between the unconventional rice G protein OsYchF1 and its well-studied activator C2-domain OsGAP1 (Fig. 1A and B). Based on sequence and structural signatures, OsYchF1 can be readily divided into the following truncated derivatives: the N-terminal core G domain (amino acids (aa) 1-128), an inserted large α-helical domain (aa 141–199), a carboxy (C)-terminal TGS domain (aa 303–394) and a No TGS (non-TGS) domain that contains both G and helical domains (aa 1-302) (Fig. 1A). The full-length *OsYchF1* and its truncated derivatives described above were constructed in pGBKT7 vectors and full-length *OsGAP1* was subcloned and inserted into the pGADT7 vector, respectively. All these combinations were co-transformed into the yeast strain AH109. Intriguingly, the combination of full-length pGBKT7-OsYchF1 with pGADT7-OsGAP1 failed to grow on synthetically defined (SD) media lacking adenine (Ade), leucine (Leu), tryptophan (Trp), and histidine (His) (SD/­Leu­Trp­His­Ade) media after 7 days at 30 °C (Fig. 1C). However, the yeast strains containing the combinations of N-terminal pGBKT7-OsYchF1 (G domain) and C-terminal pGBKT7-OsYchF1 (TGS domain) with pGADT7-OsGAP1 showed visible growth on SD/­Leu­Trp­His­Ade media (Fig. 1C).

Furthermore, the yeast strain containing the combination of pGBKT7-OsYchF1 (helical domain) with pGADT7-OsGAP1 showed no visible growth, and the combination of pGBKT7-OsYchF1 (No TGS domain) with pGADT7-OsGAP1 was also unable to grow on SD/­Leu­Trp­His­Ade media. Gal4 DNA binding domain (BD)-tagged (~ 16.2 Kda) OsYchF1 (No TGS domain) contains the G domain, and the co-presence of the helical domain appears to impair binding to Gal4 Activation Domain (AD)-tagged (~ 13.6 Kda) OsGAP1 (Fig. 1C). Taken together, these pieces of evidence suggest that OsYchF1 binds to OsGAP1 *via* the indicated N-terminal G and C-terminal TGS domains, but the inserted helical coiled-coil domain between the N- and C-terminal of OsYchF1 might partially block the interaction of BD-tagged OsYchF1 with AD-tagged OsGAP1 through steric hindrance in the Y2H system. Overall, these findings strongly imply that three relatively independent domains of OsYchF1 are evolutionarily integrated and function in an incredibly sophisticated manner.

### Thermodynamic insights into the OsYchF1–OsGAP1 complex

**Fig. 2:**
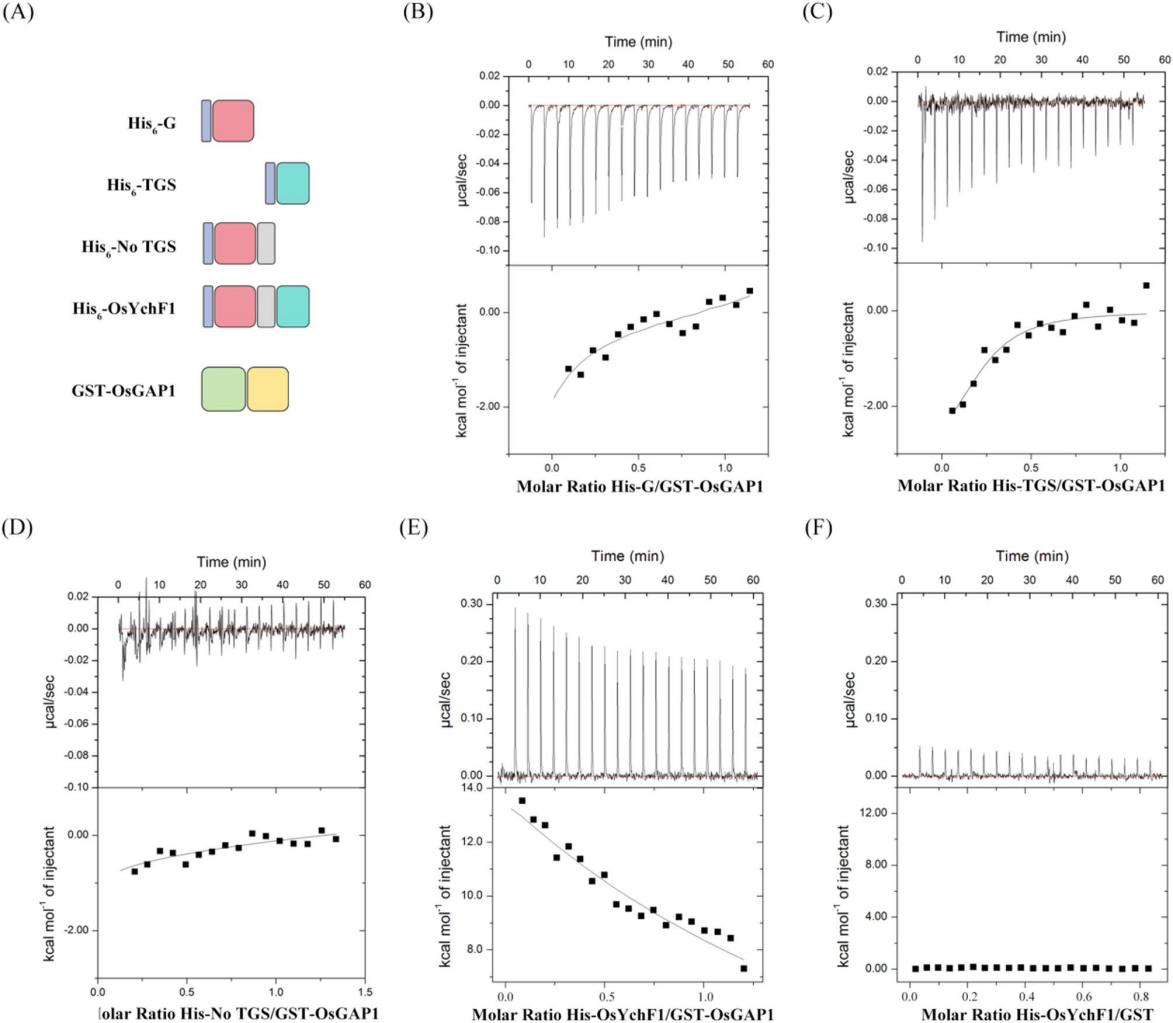
Isothermal titration calorimetry analysis of full-length OsYchF1 and its truncated derivatives with OsGAP1. (**A**) Schematic representation of His_6_-OsYchF1, its truncated derivatives and GST-OsGAP1. The His_6_-tag was color-coded in blue. The N-terminal G domain was color-coded in pink, inserted helical domain was color-coded in gray, and C-terminal TGS domain was color-coded in cyan. GST tag was color-coded in green and C2-domain OsGAP1 was color-coded in yellow. (**B**) Isothermal titration calorimetry (ITC) measurements to detect the interaction of OsYchF1 G domain with GST-OsGAP1. The binding of (His)_6_-OsYchF1 (G domain) and GST-OsGAP1 was measured by titrating 20–30 µM GST-OsGAP1 in the chamber with 400–500 µM (His)_6_-OsYchF1 (G domain) in the syringe. The interactions of GST-OsGAP1 with OsYchF1 TGS domain (**C**) and OsYchF1 No TGS domain (**D**) were measured by titrating 20–30 µM GST-OsGAP1 in the chamber with 400–500 µM (His)_6_-OsYchF1 (TGS domain) or (His)_6_-OsYchF1 (No TGS domain) in the syringe, respectively. (**E**) ITC analysis of interaction between full-length OsYchF1 and GST-OsGAP1. Binding of (His)_6_-OsYchF1 and GST-OsGAP1 was measured by titrating 20–30 µM GST-OsGAP1 in the chamber with 400–500 µM (His)_6_-OsYchF1 in the syringe. (**F**) ITC analysis of the interaction between full-length OsYchF1 and GST as a negative control. Binding of full-length OsYchF1 and GST was measured by titrating 20–30 µM GST in the chamber with 400–500 µM (His)_6_-OsYchF1 in the syringe. ITC experiments were performed at 25 ℃. Top panel, raw heating power over time; bottom panel, fit of the integrated energy values normalized for the injected protein

To further monitor the interaction of OsYchF1 with OsGAP1 in vitro, we generated N-terminal 6x His-tagged fusion proteins, including full-length (His)_6_-OsYchF1 and truncated (His)_6_-OsYchF1 (G domain) (aa 1-128), (His)_6_-OsYchF1 (TGS domain) (aa 303–394) and (His)_6_-OsYchF1 (No TGS domain) (aa 1-302) (Fig. 2A). The expression and purification of soluble recombinant full-length (His)_6_-OsYchF1, truncated (His)_6_-OsYchF1 (G domain), (His)_6_-OsYchF1 (TGS domain), (His)_6_-OsYchF1 (No TGS domain) and GST-OsGAP1 proteins were successful in *E. coli* (Fig. S1). The binding affinities of (His)_6_-tagged OsYchF1 and OsYchF1 truncated derivative proteins to the GST-tagged OsGAP1 protein were then quantified by isothermal titration calorimetry (ITC) in vitro (Fig. 2A).

The thermodynamic data demonstrated that the interaction of (His)_6_-OsYchF1 (TGS domain) with GST-OsGAP1 was reflected in an exothermic burst with a dissociation constant (*K*_D_) of 5.1 ± 2.3 µM, favored by both solvent enthalpy (Δ*H*) and entropy (*-T*Δ*S*) changes, suggesting that the binding of (His)_6_-OsYchF1 (TGS domain) to GST-OsGAP1 is strong and driven by hydrogen bonding and hydrophobic forces (Fig. 2C; Table 1). Meanwhile, the interaction of (His)_6_-OsYchF1 (G domain) with GST-OsGAP1 also showed an exothermic burst, and the *K*_D_ value was 11.9 ± 6.7 µM, suggesting that the interaction of (His)_6_-OsYchF1 (G domain) with GST-OsGAP1 is slightly weaker than that of (His)_6_-OsYchF1 (TGS domain) with GST-OsGAP1 (Fig. 2B and C; Table 1). Convincingly, there was no detectable signal in the negative controls of (His)_6_-OsYchF1 with GST-Tag and (His)_6_-tag with GST-OsGAP1 (Fig. 2F; Fig. S2; Table 1). Consistent with the results of the Y2H assay, a much weaker ITC signal was detected between (His)_6_-OsYchF1 (No TGS domain) and GST-OsGAP1 with a *K*_D_ value of 98.7 ± 24.1 (Fig. 2D; Table 1). These results confirm that the interaction of OsYchF1 with OsGAP1 in vitro depends on the N-terminal G domain and C-terminal TGS domain, and the inserted helical domain somehow interferes with the OsYchF1-OsGAP1 interaction.

**Table 1:** ITC binding constants and thermodynamic parameters for interactions of (His)_6_ tagged full-length OsYchF1, OsYchF1 G domain, OsYchF1 TGS domain and OsYchF1 no TGS domain with GST-OsGAP1

	K_D_ (µM)	*N*	ΔH	-TΔS	ΔG
			(kcal/mol)	(kcal/mol)	(kcal/mol)
(His)_6_-OsYchF1 with GST-OsGAP1	102.6 ± 11.7	0.6 ± 1.1	170.6 ± 25.8	-173.5 ± 35.6	-2.9 ± 0.3
(His)_6_-G with GST-OsGAP1	11.9 ± 6.7	0.5 ± 0.7	-2.0 ± 1.4	-0.8 ± 0.6	-2.8 ± 1.6
(His)_6_-TGS with GST-OsGAP1	5.1 ± 2.3	0.6 ± 0.1	-3.4 ± 1.1	-3.5 ± 2.5	-6.9 ± 3.3
(His)_6_-No TGS with GST-OsGAP1	98.7 ± 24.1	0.2 ± 1.0	-0.7 ± 0.1	-1.1 ± 0.4	-1.8 ± 0.2
(His)_6_-OsYchF1 with GST	N.D.	N.D.	N.D.	N.D.	N.D.
(His)_6_-tag with GST-OsGAP1	N.D.	N.D.	N.D.	N.D.	N.D.

Note: Experiments were carried out at 25 °C, and each value is the mean of three independent titrations. N.D., not detected; *K*_D_, dissociation constant; *n* value, number of binding sites (*n* = ligand/receptor); Δ*H* (enthalpy change), Δ*S* (entropy change), and Δ*G* (Gibbs free energy). The binding entropy and enthalpy determine ligand binding. Positive is unfavorable and negative is favorable (Tzeng and Kalodimos 2009, 2012)

In line with the Y2H assay, the full-length (His)_6_-OsYchF1 interacted with GST-OsGAP1 only weakly, and the binding affinity of (His)_6_-OsYchF1 to GST-OsGAP1 (*K*_D_=102.6 ± 11.7 µM) was ~ 20-fold lower than that of (His)_6_-OsYchF1 (TGS domain) to GST-OsGAP1 (*K*_D_=5.1 ± 2.3 µM) (Fig. 2C and E; Table 1). Additionally, the interaction of (His)_6_-OsYchF1 with GST-OsGAP1 was highly endothermic, favored entirely by entropy (*-T*Δ*S*) and opposed by enthalpy (Δ*H*), which usually suggests that OsYchF1 undergoes a conformational change that allows (His)_6_-OsYchF1 to expose the hydrophobic binding sites for GST-OsGAP1 (Fig. 2E; Table 1). Hence, ordered water molecules localized at the interaction surface (interface) between binding molecules are dispersed into the bulk solvent that drives the hydrophobic interaction, where water molecules are released from the OsYchF1 and OsGAP1 interface (Hariharan et al. 2015; Miao et al. 2019; Tzeng and Kalodimos 2009, 2012).

Combining the results of the Y2H and ITC experiments, the bi-terminal G and TGS domains of OsYchF1 facilitate the interaction with OsGAP1. Conversely, the inserted helical domain of OsYchF1 cannot bind to OsGAP1. Meanwhile, according to the negative control of the ITC measurements, we observed a slightly endothermic burst when (His)_6_-OsYchF1 was titrated into GST-tag (Fig. 2F), but a somewhat exothermic burst when (His)_6_-tag was titrated into GST-OsGAP1 in the negative controls (Fig. S2). The endothermic burst suggests that the GST-tag might also be involved in and interfere with the binding of GST-OsGAP1 to (His)_6_-OsYchF1. In order to bind to (His)_6_-OsYchF1, we speculate that GST-OsGAP1 must overcome the hindrance caused by its GST-tag, and the inserted helical domain and even His-tag of (His)_6_-OsYchF1 (Fig. 6). Subsequently, the exposed bi-terminal G and TGS domains tightly bound to GST-OsGAP1 via hydrogen bonding and hydrophobic interactions (Fig. 2B and C). Therefore, (His)_6_-OsYchF1 and GST-OsGAP1 are unlikely to form a stable complex. Together, these results explain why we were unsuccessful in our attempt to co-crystallize the intact OsYchF1-OsGAP1 complex.

### OsGAP1 promotes OsYchF1 enzyme activity

**Fig. 3:**
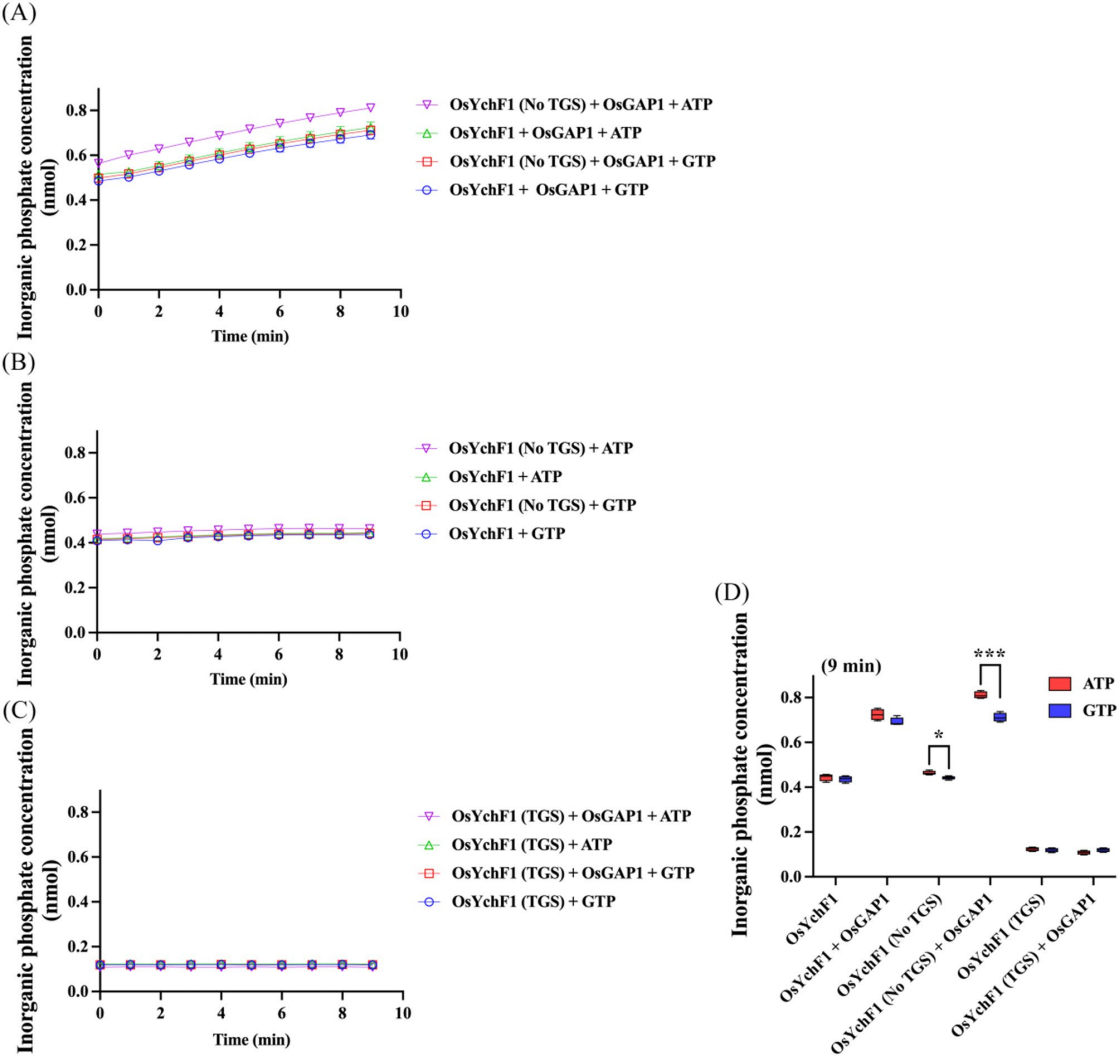
Intrinsic NTPase activity of OsYchF1 and its truncated versions in the presence of GST-OsGAP1 or GST-tag as a control. (**A**) The intrinsic NTPase activity of purified (His)_6_-OsYchF1 and its truncated derivative (His)_6_-OsYchF1 (No TGS) in the presence of GST-OsGAP1 over a 10-minute time course. (**B**) The intrinsic NTPase activity of purified (His)_6_-OsYchF1 and its truncated derivative (His)_6_-OsYchF1 (No TGS) in the presence of GST-tag as a negative control over a 10-minute time course. (**C**) The intrinsic NTPase activity of purified OsYchF1’s truncated derivative (His)_6_-OsYchF1 (TGS) in the presence of GST-OsGAP1 or GST-tag over a 10-minute time course. (**D**) The intrinsic NTPase activity of purified OsYchF1 and its truncated derivatives in the presence of GST-OsGAP1 or GST-tag after 9-minute. GST-tag was used as a control. The molar ratio of GST-OsGAP1/(His)_6_-OsYchF1 and its truncated derivatives was ~ 1–2. Error bars mean the SD; *n* = 12

Subsequently, we measured the NTPase activity of (His)_6_-OsYchF1 and its (His)_6_-tagged truncated derivatives in the absence or presence of GST-OsGAP1 over a 10-minute time course. Both (His)_6_-OsYchF1 and (His)_6_-OsYchF1 (No TGS domain) including G and helical domains displayed similar GTPase and ATPase activity compared to (His)_6_-OsYchF1 (TGS domain) without the enzymatic G domain as a negative control (Fig. 3B and C). Meanwhile, GST-OsGAP1 noticeably promoted the NTPase activity of (His)_6_-OsYchF1 as well as (His)_6_-OsYchF1 (No TGS domain) (Fig. 3A). However, (His)_6_-OsYchF1 (No TGS domain) exhibited higher ATPase activity than GTPase activity in the absence or presence of GST-OsGAP1 (Fig. 3A, B and D), indicating that the TGS domain may limit the full capability of OsYchF1 ATPase activity. Taken together, although the interaction of OsYchF1 (No TGS domain) with OsGAP1 was barely observed in Y2H and ITC measurements, (His)_6_-OsYchF1 (No TGS domain) still maintained GST-OsGAP1-stimulated NTPase activity, which implies that OsGAP1 might transiently interact with OsYchF1.

### AlphaFold2 fails to predict accurate structural models of the OsYchF1–OsGAP1 complex

Proteins form complexes to perform their biological functions. The crystal structure of the complex is critical for understanding the molecular mechanisms, but many of them are quite difficult to co-crystallize. The Google DeepMind and EMBL-EBI developed AI system AlphaFold2 for protein structure prediction is believed to be crucial in speeding up scientific research. However, the validity of AlphaFold2 structure prediction for weak protein-protein interactions remains largely unknown. Herein, we fully exploited the universally conserved rice unconventional G-protein OsYchF1 and its C2-domain activator OsGAP1 as an instance of weak protein-protein interaction (*K*_D_=102.6 ± 11.7 µM) to test the effects of AlphaFold2 structure prediction on weak protein complexes.

**Fig. 4:**
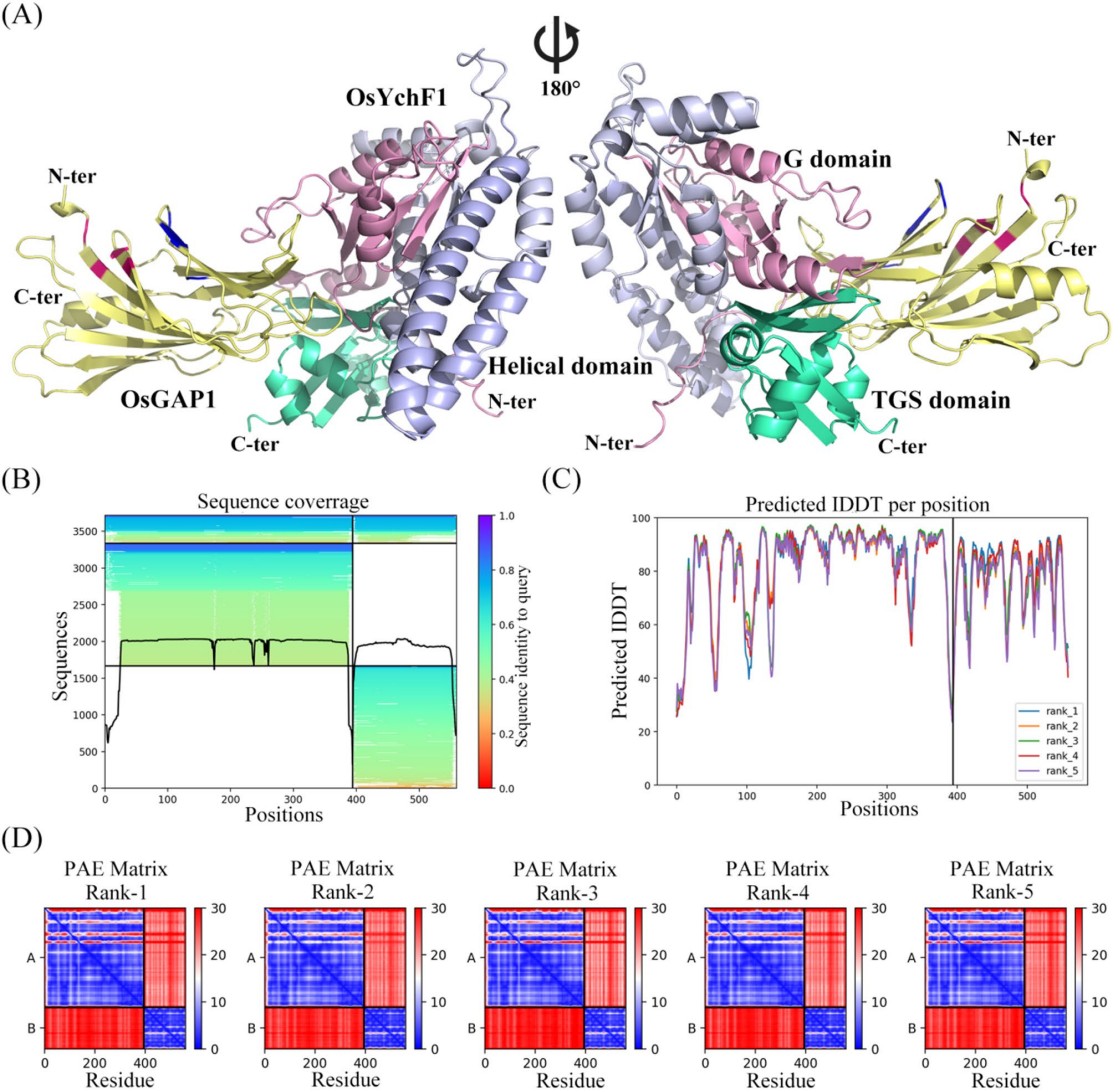
AlphaFold2 predicts the structural model of the OsYchF1–OsGAP1 complex. (**A**) The top-ranked model of the OsYchF1–OsGAP1 complex. N-terminal core G domain is pink; inserted α-helical domain is lightblue; C-terminal TGS domain is cyan; C2-domain OsGAP1 is yellow. Architectural and training details of the OsYchF1–OsGAP1 complex. The indicated cluster 1 and 3 were color-coded in blue and red, respectively. (**B**) The sequence identity to the query of the OsYchF1–OsGAP1 complex. (**C**) The predicted Local Distance Difference Test (pIDDT) per position for the OsYchF1–OsGAP1 interface. (**D**) Predicted Aligned Error (PAE) matrix for the prediction of the OsYchF1–OsGAP1 complex. The black lines indicate the chain boundaries. Blue and red gradients indicate PAE and darker blue is more confident. The side bar means the expected positional error (Å)

We ran AlphaFold2 five times to predict the OsYchF1–OsGAP1 complex structure. Because the high-resolution crystal structures of OsYchF1 and OsGAP1 were resolved, each workflow involved the prediction of five accurate three-dimensional structural models of the OsYchF1–OsGAP1 complex (Fig. 4A). First, the input primary sequence of the OsYchF1–OsGAP1 complex showed high identity to the genetic sequence databases (Fig. 4B). Second, the quality of the prediction was evaluated by measuring the predicted Local Distance Difference Test (pLDDT; from 0 (minimum value; being the worst) to 100 (maximum value; being the best), representing the distances of all heavy atoms across various chains in the protein–protein interface (Mariani et al. 2013). Almost similar pLDDT was observed for all predictions (Fig. 4C). Third, the Predicted Aligned Error (PAE) matrix, indicating the confidence in the positioning between the predicted residues and the true structures, was stable for these predicted models (Fig. 4D).

Interestingly, all predicted models demonstrated that OsYchF1 interacts with OsGAP1 *via* the bi-terminal G domain and TGS domain, resembling the experimental results described above (Fig. 4A). We selected and exhibited the top-ranked model with an excellent pLDDT score of 81.7 (Fig. 4A). The pLDDT score is an important metric for evaluating the confidence in predicted protein structures. A pLDDT score greater than 80 usually indicates a predicted structure with high confidence (Bryant et al. 2022; Guo et al. 2022). However, a previous study showed that OsGAP1 interacts with OsYchF1 through cluster 1 (Leu-5, Leu-8, Thr-58 and Ser-60) and cluster 3 (Lys-37, Lys-39, Lys-40 and Arg-43) locating on the same side of the OsGAP1 protein surface (Fig. 2B) (Yung et al. 2015). Unfortunately, the residues in clusters 1 and 3 are far away from the OsYchF1 binding site in the AlphaFold2 model shown in Fig. 4A. This indicates that Alphafold2 is not a promising tool for modelling this weak protein-protein interaction in the OsGAP1-OsYchF1 complex.

### ClusPro generates highly accurate structural models of the OsYchF1–OsGAP1 complex

**Fig. 5:**
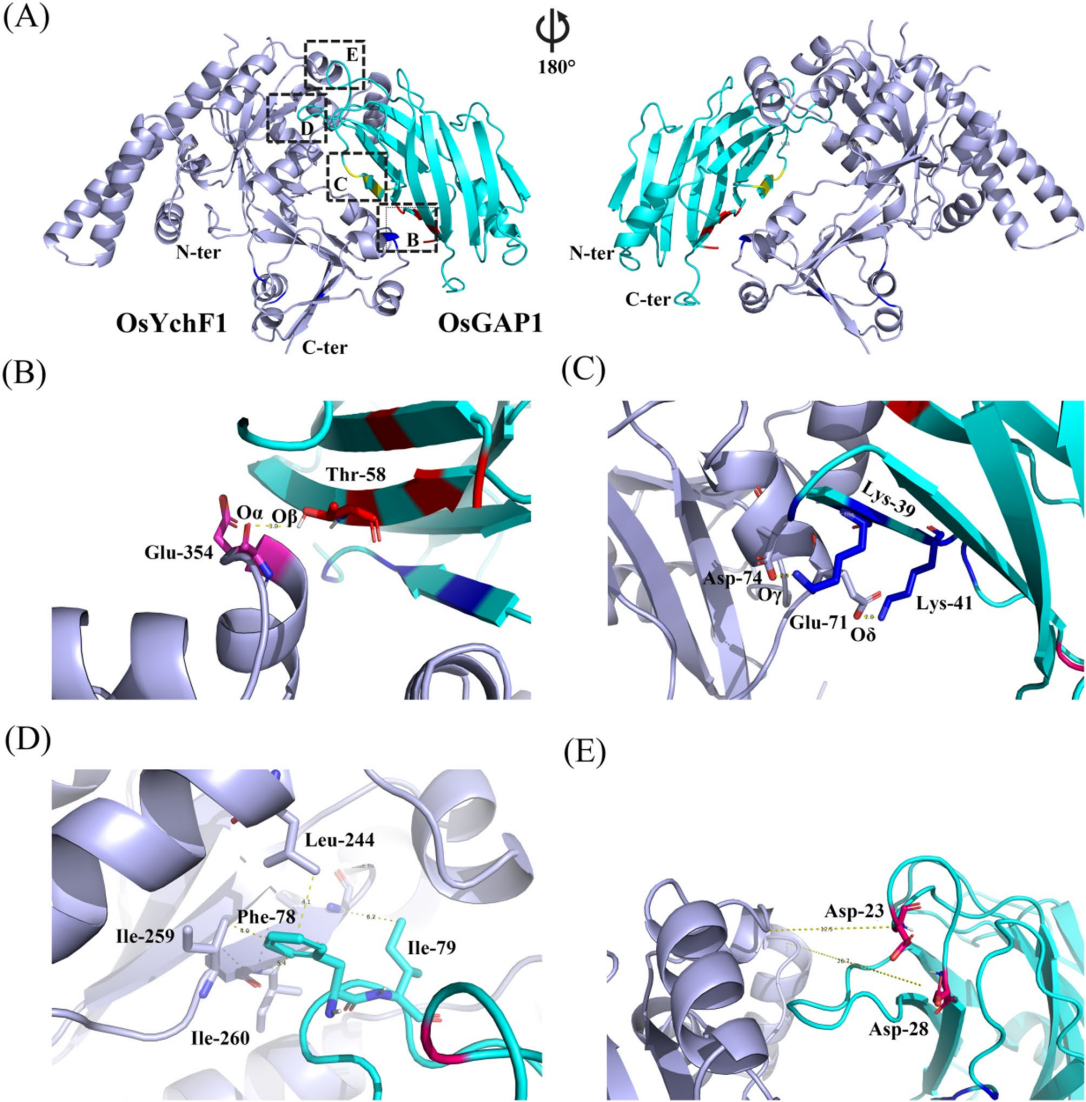
ClusPro creates accurate OsYchF1–OsGAP1 docking models. (**A**) The top-ranked model of the OsYchF1–OsGAP1 complex produced by ClusPro 2.0. OsYchF1 was color-coded in lightblue, and OsGAP1 was color-coded in cyan. The indicated clusters 1 and 3 were color-coded in yellow and red, respectively. The four critical amino acid residues in the TGS domain of OsYchF1 were color-coded in blue. (**B**) The specific amino acid residue Glu-354 located at the OsYchF1 TGS domain are implicated in the interaction of OsYchF1 with the Thr-58 residue located at the cluster region 3. (**C**) The Glu-71 and Asp-74 residues located at the G domain form salt bridge with the Lys-41 and Lys-39 residues at the cluster 3 of OsGAP1 respectively. (**D**) The Phe-78 and ILe-79 non-polar amino acid residues of OsGAP1 bind to the hydrophobic pocket of OsYchF1. (**E**) The cluster 3 (Asp-23 and Asp-28) of OsGAP1 are not involved in the interaction of OsGAP1 with OsYchF1. The distance unit is Angstrom (Å)

Because the crystal structures of both OsYchF1 and OsGAP1 were determined experimentally. We decided to use template-based docking programs (e.g., ClusPro) to predict the complex structure instead of using AlphaFold2. Much to our surprise, ClusPro 2.0 generates a series of accurate structural models of the OsYchF1–OsGAP1 complex (Fig. 5A). A previous study reported that four amino acid residues (Lys-325, His-334, Glu-345 and Glu-354) on the surface of the OsYchF1 C-terminal TGS domain are critical for the interaction between OsYchF1 and OsGAP1 according to site-directed mutagenesis analyses (Cheung et al. 2020). In line with the previous results, more specifically, the Oα at the backbone of Glu-354 located at the OsYchF1 TGS domain forms a hydrogen bond with the Oβ located at the side-chain of Thr-58 of OsGAP1 (Fig. 5B).

Meanwhile, according to the ClusPro-generated OsYchF1-OsGAP1 docking model, the negatively charged Glu-71 located at the G domain of OsYchF1 and the positively charged Lys-41 at the OsGAP1 cluster 3 formed a salt bridge, while another salt bridge was formed between Asp-74 at the G domain of OsYchF1 and Lys-39 at the OsGAP1 cluster 3 (Fig. 5C). Intriguingly, the hydrophobic amino acid residues Phe-78 and Ile-79 on OsGAP1 was shown to anchor to the hydrophobic pocket of the non-polar amino acid residues Leu-244, Ile-259 Ile-260 and Phe-262 on OsYchF1 (Fig. 5D), which is in agreement with the ITC data (Fig. 2E; Table 1).

In addition, Yung et al. (2015) documented that cluster region 2 (Asp-23 and Asp-28) is not involved in the interaction of OsYchF1 with OsGAP1. As shown in Fig. 5E, the Asp-23 and Asp-28 located in cluster 2 of OsGAP1 are far away from OsYchF1 indeed. In summary, AlphaFold2 cannot replace X-ray crystallography as it does not generate a promising protein-protein docking model (Fig. 4). However, template-based docking programs (e.g., ClusPro) combined with wet-lab experimental evidence facilitate the prediction of protein-protein interactions and benefit in studying the function of protein complexes that rarely co-crystallize.

## Discussion

The YchF subfamily comprises a group of highly conserved P-loop NTPases that bind and hydrolyze both GTP and ATP, and are associated with critical cellular processes from bacteria to mammals, such as oncogenesis, protein synthesis and stress response (Balasingam et al. 2019). Our previous work has shown that OsGAP1 binds to OsYchF1 and accelerates OsYchF1 NTPase activity (Yung et al. 2015). In the present study, the in vitro thermodynamic data obtained by ITC are in line with the results of the Y2H assay, and confirm the hypothesis that the N- and C-terminal G and TGS domains of OsYchF1 may facilitate the binding of OsYchF1 to OsGAP1. On the other hand, the data collected from the designed truncated system are not only consistent with those from the previous study (Cheung et al. 2020), proving the reliability of our designed truncated system, but also provide new incoming thermodynamic information. OsYchF1 interacts with OsGAP1 *via* its G and TGS domains, yet the helical domain sterically hinders this interaction in our experimental system.

The crystal structures of protein complexes are critical for understanding the biological functions and molecular mechanisms. However, the low-binding affinity protein complexes barely achieve co-crystallization for structural determination. Recent advances in deep-learning-based structure prediction enable accurate modeling of protein complexes, and facilitate scientific research to understand the biological function of protein complexes (Humphreys et al. 2021), yet the validity of the structure prediction model for weak protein-protein interactions keeps largely unknown from the AlphaFold2. Therefore, we ran AlphaFold2 to predict the three-dimensional structure of the OsYchF1–OsGAP1 complex, which represents a typical weak protein-protein interaction (*K*_D_=102.6 ± 11.7 µM), and this complex cannot be co-crystallized at all.

Using the OsYchF1–OsGAP1 complex as an example, more importantly, we found that a main limitation of AlphaFold2 structure prediction is that AlphaFold2 generates a relatively accurate structure of protein complexes with high binding affinity. The N-terminal G domain and the C-terminal TGS domain of OsYchF1 bind tightly to OsGAP1 with similar *K*_D_ values (11.9 ± 6.7 µM and 5.1 ± 2.3 µM), both in the micromolar range, driven equally by the hydrogen bonding (enthalpy; -2.0 ± 1.4 kcal/mol and − 3.4 ± 1.1 kcal/mol) and the hydrophobic forces (entropy; -0.8 ± 0.6 kcal/mol and − 3.5 ± 2.5 kcal/mol). This aligns with previous studies at least partly (Cheung et al. 2020), yet raises questions about the role of the helical domain in modulating affinity.

Notably, AlphaFold2 failed to model the dynamic conformational changes of the OsYchF1-OsGAP1 complex, likely due to its transient binding nature. The binding of intact OsYchF1 to OsGAP1 was highly exothermic with a weak binding affinity (102.6 ± 11.7 µM), occurring in the submillimolar range. This interaction was exclusively driven by the hydrophobic interaction (entropy; *-T*Δ*S*=-173.5 ± 35.6 kcal/mol), contrasting with hydrogen bonding (enthalpy; Δ*H* = 170.6 ± 25.8 kcal/mol). Therefore, the predicted protein-protein docking models are inconsistent with previous experimental results (Yung et al. 2015). This limitation highlights that AlphaFold2 remains inadequate for predicting weak protein-protein interactions.

**Fig. 6:**
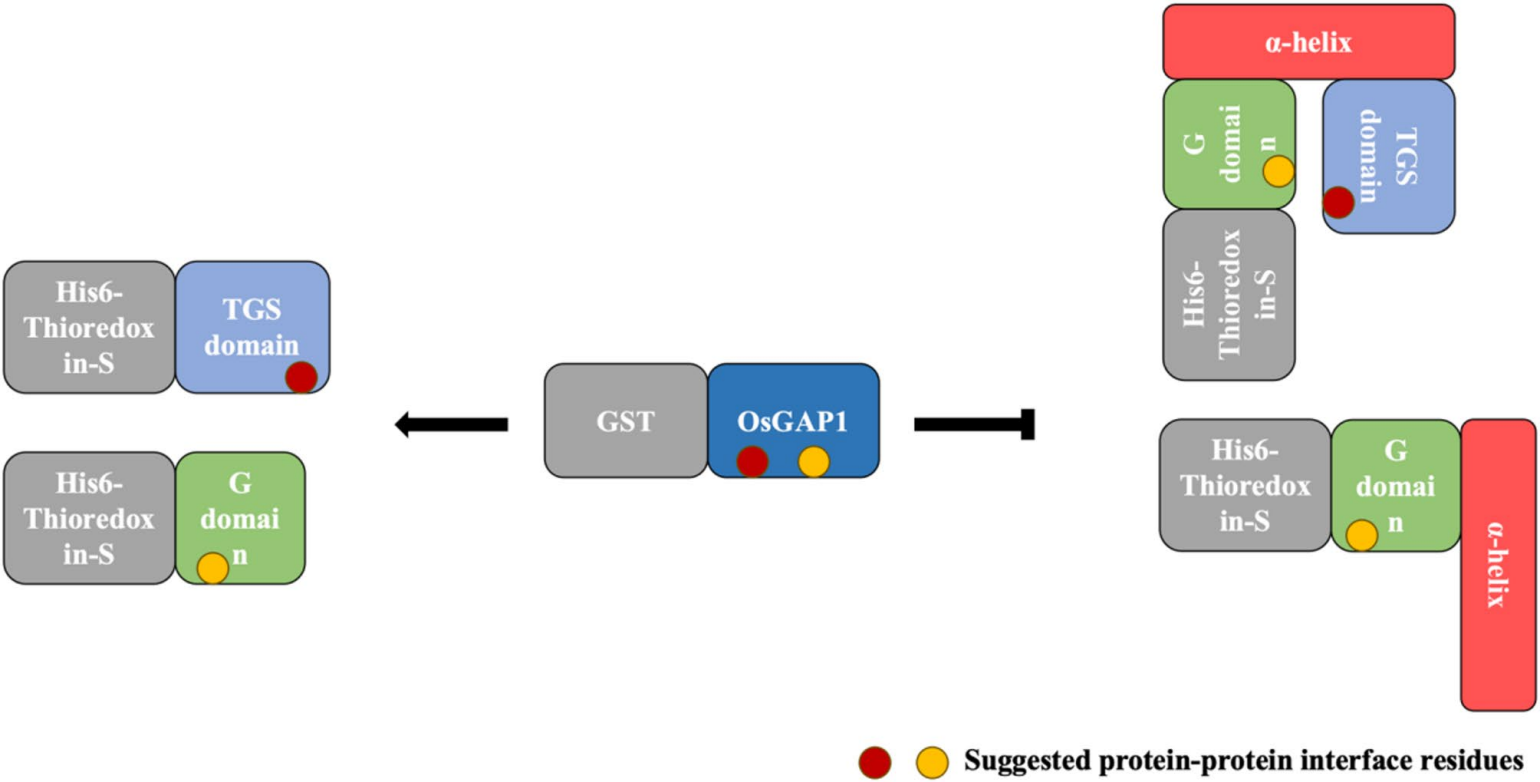
Suggested model to explain the possible interactions of GST-OsGAP1 with (His)_6_-OsYchF1 and its truncated versions. The (His)_6_-tagged (~ 15 kDa) OsYchF1 G and TGS domains bind to the GST-tagged (~ 26 kDa) OsGAP1. However, (His)_6_-OsYchF1 and (His)_6_-OsYchF1 No TGS domain cannot interact with GST-OsGAP1 due to a steric hindrance. OsYchF1 and its truncated versions were cloned into a pET28a expression vector that contains a hexahistidine tag, Thioredox (Trx) and an S-tag at the N-terminus of the fusion protein

Moreover, we confirm the reliability of template-based docking programs (e.g., ClusPro). The protein-protein docking by a fully automated algorithm ClusPro is not only consistent with the experimental evidence that the G and TGS domains of OsYchF1 facilitate the interaction with OsGAP1, but also with the previous results that the specific cluster regions on OsGAP1 are implicated in the binding of OsGAP1 to OsYchF1 (Fig. 6). In addition to this work, Araujo-Abad et al. (2023; 2024) predicted the citrullinating enzyme PADI4 (PROTEIN-ARGININE DEIMINASE 4)-epigenetic factor RYBP (RING1 AND YY1 BINDING PROTEIN) complex and PADI4 with the two fragments of the transcriptional repressor C-RING1B and N-RING1B complex in human cancer cells by using the protein-protein docking algorithm ClusPro. In conclusion, based on the experimental evidence, ClusPro can provide accurate protein-protein docking models for future studies on weak protein-protein complexes, but not AlphaFold2.

## Experimental procedures

### Yeast two-hybrid assay

The full-length *OsGAP1* coding sequence was subcloned into pGADT7 (*Nde*I and *BamH*I) vector, and the coding sequences of full-length *OsYchF1* and *OsYchF1* truncated versions were subcloned into pGBKT7 (*EcoR*I and *Sal*I) vector. Yeast two-hybrid (Y2H) assays were conducted following the Matchmaker Gold Yeast Two­Hybrid System manual (Clontech) with a minor modification. All combinations were co-transformed into yeast strain AH109, and then eventually grown on SD (synthetically defined)/-Lue/­Trp/­His/­Ade plates to screen for protein-protein interactions (Miao et al. 2021). All primers used for plasmid construction are listed in Table S1.

### Protein expression and purification

The digested fragments of full-length *OsYchF1* and its truncated derivatives were inserted into pET-32a (*EcoR*I and *Hind*III) expression vector by using T4 DNA ligase. The digested fragment of the full-length *OsGAP1* coding sequences was inserted into pGEX-4T-1 (*BamH*I and *Xho*I) expression vector. The plasmids were used for *Escherichia. coli* (*E. coli*) DH5α transformation. Transformants containing the *OsYchF1* and *OsGAP1* constructs were screened by colony PCR using the T7/T7 terminator and pGEX5’/pGEX3’ primer pairs, respectively. All constructs were verified by sanger DNA sequencing across the cloning sites to ensure in-frame N-terminal 6x polyhistidine (His_6_) tagged or glutathione S-transferase (GST) tagged fusion proteins. All primers used for plasmid construction are listed in Table S1. *E. coli* BL21 (DE3) pLysS competent cells were transformed with the expression vectors containing full-length *OsYchF1* and its truncated derivatives, full-length *OsGAP1*. Protein expression and purification were carried out as previously reported (Miao et al. 2019).

### Isothermal titration calorimetry (ITC) measurements

The interactions of full-length (His)_6_-OsYchF1 and its truncated derivatives including (His)_6_-OsYchF1 (G domain), (His)_6_-OsYchF1 (TGS domain) and (His)_6_-OsYchF1 (No TGS domain) with GST-OsGAP1 or GST-tag were analyzed using a MicroCal ITC200 system (Malvern Panalytical) as described previously (Miao et al. 2019). Prior to ITC measurements, all proteins were dialyzed against a dialysis buffer: 20 mM Tris-HCl pH = 7.5, 20 mM NaCl and 1 mM DTT at 4 ℃ overnight. 400–500 µM full-length (His)_6_-OsYchF1, (His)_6_-OsYchF1 (G domain), (His)_6_-OsYchF1 (TGS domain), (His)_6_-OsYchF1 (No TGS domain) and (His)_6_-tag in the syringe were titrated to 20–30 µM GST-OsGAP1 or GST-tag in the chamber. The ITC experiments were repeated twice with similar results at 25 ℃.

### P-loop NTPase activity assay

The P-loop NTPase activity of 20–30 µM purified (His)_6_-OsYchF1 and its truncated derivatives including (His)_6_-OsYchF1 (No TGS domain) and (His)_6_-OsYchF1 (TGS domain) in the absence or presence of 20–30 µM GST-OsGAP1 or 20–30 µM GST Tag as a control was measured by determining the release of inorganic phosphate (Pi) during 0.5 mM GTP and 0.5 mM ATP hydrolysis using the EnzChek™ Phosphate Assay Kit (E6646; Molecular Probes, Carlsbad, CA, USA) according to a previous method (Cheung et al. 2008).

### Prediction of OsYchF1–OsGAP1 structures by AlphaFold2

The prediction of the OsYchF1–OsGAP1 structure was performed using the AlphaFold2 fulfillment in the ColabFold notebooks implemented on Google Colaboratory (Tunyasuvunakool et al. 2021; Jumper et al. 2021; Mirdita and Ovchinnikov, 2021). The default settings with Amber relaxation were listed below according to Fontana et al. (2022). msa method: mmsegs2homooligomer-l, subsample msa: True, num relax: 5, pair mode: unpaired, max msa-512: 1024, use ptm: True, use turbo: True, rank by pLDDT, num models: 5, num samples: 1, num ensemble: 1, max recycles: 5, tol: 0, is training: False, use templates: False. The major difference between AlphaFold2 fulfillment and ColabFold is that ColabFold’s uses mmsegs2 (Steinegger and Soding et al. 2017), which the authors of the ColabFold announcement generate fair data (Mirdita and Ovchinnikov, 2021). For the prediction of the OsYchF1–OsGAP1 complex, the OsYchF1 and OsGAP1 sequences were entered in tandem and separated by a semicolon. The predicted Protein Data Bank (PDB) files produced by AlphaFold2 were then imported into PyMOL to evaluate the interaction regions. The rank-1 file with the highest pLDDT score was picked for further analysis. Specific commands were used to separate and colour the two sequences, OsYchF1 and OsGAP1. An additional Python script (InterfaceResidues.py) was then executed to identify and label the interacting amino acid residues. The top-rank structural model generated for the OsYchF1 and OsGAP1 complex has been deposited in the ModelArchive repository at (https://www.modelarchive.org/doi/10.5452/ma-n6brr).

### ClusPro docking

We utilized the webserver http://newbiophysics.cs.vt.edu/H++/ to compute the protonation states of ionizable groups in OsYchF1 and OsGAP1 under the ITC measurement conditions (salinity: 20 mM; internal dielectric 8; external dielectric 79; pH: 7.5) (Gordon et al. 2005). The crystal structures of OsYchF1 (PDB code: 5EE1) and OsGAP1 (PDB code: 4RJ9) were inputted as the templates. Then, the OsGAP1-OsYchF1 docking model was produced using the template-based docking web server ClusPro 2.0 (https://cluspro.org) (Kozakov et al. 2017; Desta et al. 2020). The ClusPro server automatically performed the rigid-body docking algorithms base on fast Fourier transform (FFT) correlation techniques. The output protein-protein docking structures were a shortlist of energetically favourable structures based on a pairwise binding site root mean squared deviation (RMSD) criterion.

## Supplementary Information

Below is the link to the electronic supplementary material.

Supplementary Material 1

Supplementary Material 2

## Data Availability

Correspondence and requests for materials should be addressed to Dr. Rui Miao.
