# Supplementary material for: From biophysical interaction to structural modeling: bi-terminal G and TGS domains drive rice OsYchF1-OsGAP1 complex formation
Source: Bot Stud. 2025 Sep 29;66:29. doi: 10.1186/s40529-025-00480-0 (PMC12480156; doi:10.1186/s40529-025-00480-0)
Supplement: Supplementary file 1 — Supplementary Material 1 [file 40529_2025_480_MOESM1_ESM.docx]

**Table S1. Primers used in the study.**

| Primer name **Sequence 5’→3’** |
| --- |
| pGADT7-OsGAP1-F AACATATGATGTTGGGGCATCTGGTTGG  pGADT7-OsGAP1-R CCCGGATCCTCATACACCCTTAGAACCTGGGATG  pGBKT7-OsYchF1-F AAGAATTCATGCCGCCCAAGGCGTC  pGBKT7-OsYchF1-R CCCGTCGACCTTCTTTCCACCTCCAG  GST-OsGAP1-F AAGGATCCATGTTGGGGCATCTGGTTGGGC  GST-OsGAP1-R CCCCTCGAGTCATACACCCTTAGAACCTGGGATGTCG  His-OsYchF1-F AAGAATTCATGCCGCCCAAGGCGTC  His-OsYchF1-R CCCAAGCTTCTTCTTTCCACCTCCAG  His-OsYchF1(G)-R CCCAAGCTTTCATGCTCTCAATACATGAAAAATTCCATCAACAGC  His-OsYchF1(No TGS)-R CCCAAGCTTATGGATTGCTGCAAAACCAGTCTTG  His-OsYchF1(Helical)-F AAGAATTCATGGTGGATCCTGTTAGAGATTTGGAAACTATTGG  His-OsYchF1(Helical)- R CCCAAGCTTCTTTCCATCTTCAAGATGGGCTTTGACCTTC  His-OsYchF1(TGS)-F AAGAATTCATGCTTATATACTTTTTCACTGCTGGCCCTGAC  His-OsYchF1(TGS)-R CCCAAGCTTCTTCTTTCCACCTCCAGACAC  pGBKT7- G-R AGAGCTCTCATGCTCTCAATACATGAAAAATTCCATCAACAGC  pGBKT7-No TGS-R AAAGAGCTCTCAATGGATTGCTGCAAAACCAGTCTTG  pGBKT7-α-F CCGAATTCATGGTGGATCCTGTTAGAGATTTGGAAACTATTGG  pGBKT7-α-R AAAGAGCTCTCACTTTCCATCTTCAAGATGGGCTTTGAC  pGBKT7-TGS-F CCGAATTCATGCTTATATACTTTTTCACTGCTGGCCCTGAC  pGBKT7-TGS-R AAAGAGCTCTCACTTCTTTCCACCTCCAGACAC |
