# Supplementary material for: From biophysical interaction to structural modeling: bi-terminal G and TGS domains drive rice OsYchF1-OsGAP1 complex formation
Source: Bot Stud. 2025 Sep 29;66:29. doi: 10.1186/s40529-025-00480-0 (PMC12480156; doi:10.1186/s40529-025-00480-0)
Supplement: Supplementary file 2 — Supplementary Material 2 [file 40529_2025_480_MOESM2_ESM.docx]

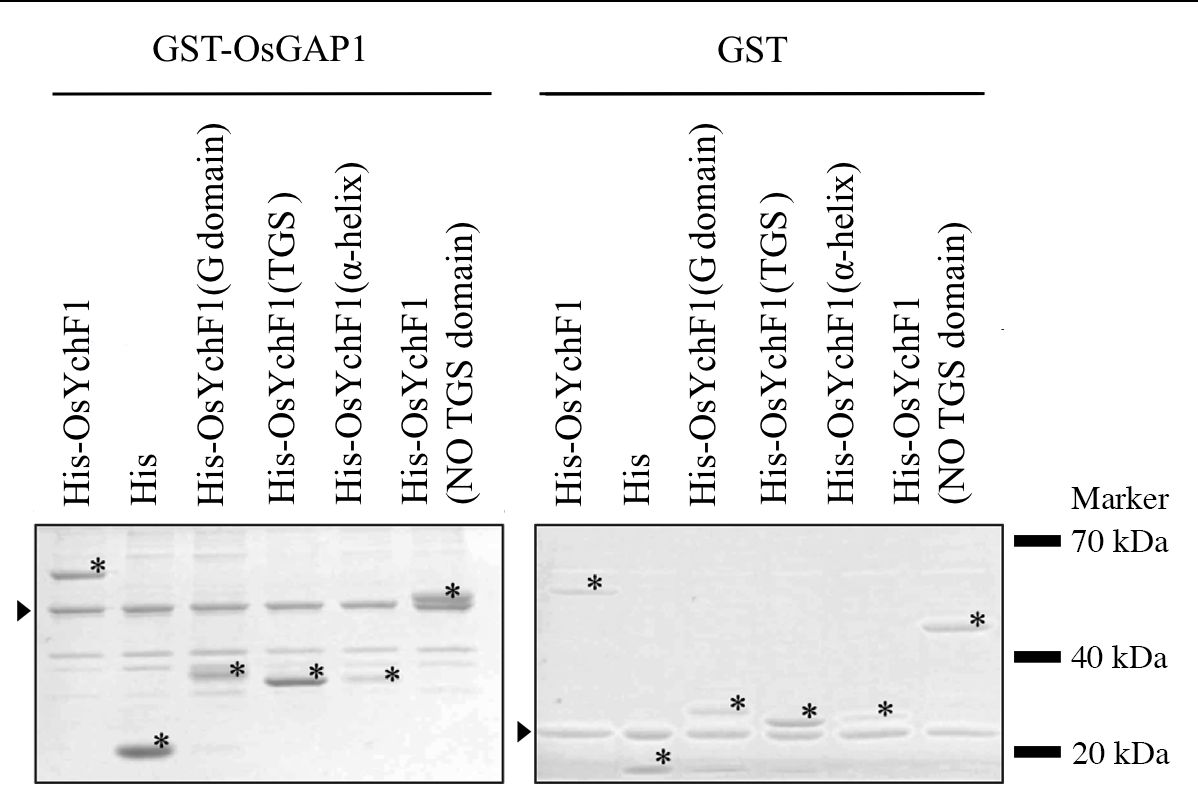


**Figure S1. The expression levels of intact and truncated OsYchF1 with GST-OsGAP1 and GST tag.** The expression and purification of soluble recombinant full-length (His)_6_-OsYchF1, truncated (His)_6_-OsYchF1 (G domain), (His)_6_-OsYchF1 (helical domain), (His)_6_-OsYchF1 (TGS domain), (His)_6_-OsYchF1 (No TGS domain) and GST-OsGAP1 proteins are shown. Asterisks indicate the recombinant full-length (His)_6_-OsYchF1, truncated (His)_6_-OsYchF1 (G domain), (His)_6_-OsYchF1 (helical domain), (His)_6_-OsYchF1 (TGS domain), (His)_6_-OsYchF1 (No TGS domain) and (His)_6_ tag. Triangle symbols indicate the recombinant GST-OsGAP1 and GST tag.


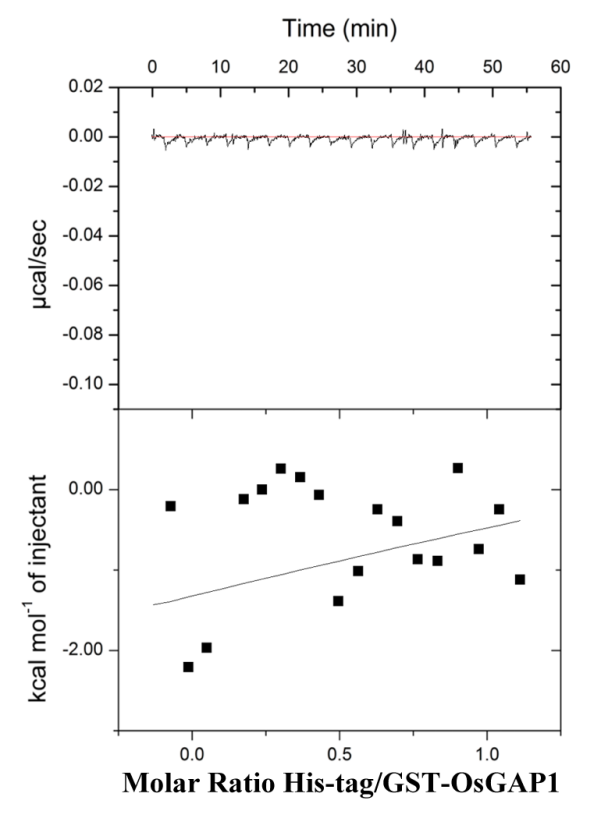


**Figure S2. Isothermal titration calorimetry analysis of (His)_6_-tag with GST-OsGAP1.** Isothermal titration calorimetry (ITC) measurements to detect the interaction of (His)_6_-tag with GST-OsGAP1. The binding of (His)_6_-tag and GST-OsGAP1 was measured by titrating 20-30 μM GST-OsGAP1 in the chamber with 400-500 μM (His)_6_-tag in the syringe. ITC experiments were performed at 25 ℃. Top panel, raw heating power over time; bottom panel, fit of the integrated energy values normalized for the injected protein. The ITC experiments were repeated twice with similar results at 25 ℃.
